# Supplementary material for: Antigenic mapping of the hemagglutinin of the H9 subtype influenza A viruses using sera from Japanese quail (Coturnix c. japonica)
Source: J Virol. 2023 Oct 6;97(10):e00743-23. doi: 10.1128/jvi.00743-23 (PMC10617583; doi:10.1128/jvi.00743-23)
Supplement: Table S1 — Homologous HI titers against chimeric HA-WF10 (H9N2) viruses using individual quail sera after prime. [file jvi.00743-23-s0001.docx]

**Table S1**. Homologous HI titers against chimeric HA-WF10 (H9N2) viruses using individual quail sera after prime.

|  | **Quail** **Sera** | | | | | | | | | | | | | | | | | |  |
| --- | --- | --- | --- | --- | --- | --- | --- | --- | --- | --- | --- | --- | --- | --- | --- | --- | --- | --- | --- |
| **Virus** | | **h9.1.1** | | | | | | **h9.2.2** | | | | | | **h9.4.2** | | | | | |
| **Serum sample** | | **1** | **2** | **3** | **4** | **5** | **6** | **1** | **2** | **3** | **4** | **5** | **6** | **1** | **2** | **3** | **4** | **5** | **6** |
| **Homologous HI titer** | | <10 | <10 | <10 | <10 | <10 | <10 | <10 | <10 | 40 | <10 | <10 | <10 | 640 | 640 | 640 | 640 | 320 | 320 |
| **Virus** | | **h9.3.3** | | | | | | **h9.3.4** | | | | | | **h9.3.5** | | | | | |
| **Serum sample** | | **1** | **2** | **3** | **4** | **5** | **6** | **1** | **2** | **3** | **4** | **5** | **6** | **1** | **2** | **3** | **4** | **5** | **6** |
| **Homologous HI titer** | | <10 | 160 | <10 | 160 | 20 | ND | <10 | 160 | <10 | 640 | 320 | 160 | 80 | 80 | <10 | <10 | 80 | 40 |
| **Virus** | | **h9.3.7** | | | | | | **h9.3.9** | | | | | | **WF10 (h9.4.1)** | | | | | |
| **Serum sample** | | **1** | **2** | **3** | **4** | **5** | **6** | **1** | **2** | **3** | **4** | **5** | **6** | **1** | **2** | **3** | **4** | **5** | **6** |
| **Homologous HI titer** | | 80 | 160 | 640 | 160 | 320 | 40 | 40 | 640 | 160 | 160 | 320 | 640 | 640 | 640 | 640 | 640 | 320 | 320 |
