# Supplementary material for: Antigenic mapping of the hemagglutinin of the H9 subtype influenza A viruses using sera from Japanese quail (Coturnix c. japonica)
Source: J Virol. 2023 Oct 6;97(10):e00743-23. doi: 10.1128/jvi.00743-23 (PMC10617583; doi:10.1128/jvi.00743-23)
Supplement: Table S2 — ACMACS HI titers of the different H9 viruses using quail sera raised against chimeric H9N2 viruses. [file jvi.00743-23-s0002.docx]

**Table S2.** ACMACS HI titers of the different H9 viruses using quail sera raised against chimeric H9N2 viruses.
